# Beyond race: Impacts of non-racial perceived discrimination on health access and outcomes in New York City

**DOI:** 10.1371/journal.pone.0239482

**Published:** 2020-09-24

**Authors:** Prabal K. De

**Affiliations:** Department of Economics and Business, City College, City University of New York, New York, NY, United States of America; Università degli Studi di Perugia, ITALY

## Abstract

**Background:**

I investigate the association of perceived discrimination based both on race and other attributes such as age, gender, and insurance status on self-reported health access and health outcomes in a diverse and densely populated metropolitan area.

**Methods:**

Restricted data from the 2016 round of the New York City Community Health Survey was used to create prevalence estimates for both racial and non-racial discrimination. Logistic regression models were used to estimate the association of these discrimination measures with health access and health outcome variables.

**Results:**

Among residents who perceived discrimination receiving health care during the previous year, 15% reported the reason behind such discrimination to race, while the rest chose other reasons. Among the non-race based categories, 34% reported the reason behind such discrimination to be insurance status, followed by other reasons (26.83%) and income (11.76%). Non-racial discrimination was significantly associated with the adjusted odds of not receiving care when needed (AOR = 6.96; CI: [5.00 9.70]), and seeking informal care (AOR = 2.24; CI: [1.13 4.48] respectively, after adjusting for insurance status, age, gender, marital status, race/ethnicity, nativity, and poverty. It was also associated with higher adjusted odds of reporting poor health (AOR = 2.49; CI: [1.65 3.75]) and being diagnosed with hypertension (AOR = 1.75; CI: [1.21 2.52]), and diabetes (AOR = 1.84; CI: [1.22 2.77]) respectively.

**Conclusions:**

Perceived discrimination in health care exists in multiple forms. Non-racial discrimination was strongly associated with worse health access and outcomes, and such experiences may contribute to health disparities between different socioeconomic groups.

## Introduction

Disparities in both mental and physical health outcomes between various population groups have long been a concern among health researchers and policymakers [1, 2]. There is growing evidence that experiences of discrimination in health care settings may contribute to such disparities. Perceived discrimination broadly refers to the situation when an individual perceives to be treated in an inferior way compared to other socioeconomic groups. Previous research has found such discrimination to be one of the factors mediating the relationship between group membership and health outcomes [3–9]. Given the nature of disparities in health access and outcomes in the United States, where historically African-Americans have shown worse access and outcomes across various indicators, this literature has primarily focused on race/ethnicity-based discrimination, particularly between whites and African Americans [10–12]. In recent years, discrimination research has expanded its scope and found evidence of an association between perceived discrimination and adverse health outcomes among other minority groups also, such as Asian Americans and Hispanic Americans [13, 14].

However, patients in a medical setting can potentially perceive some institutional or interpersonal behaviors as discriminatory owing to several personal attributes, not just race/ethnicity. Subsequently, providers, researchers, and policymakers have come to understand that *any* group identity based on gender, gender orientation, immigration status, insurance status, income, body weight can be at risk of perceiving discrimination in health care settings [15, 16]. Specifically, studies have investigated the negative impacts of discrimination in health care based on socioeconomic status [1, 17], age [18], body weight [19–22], and gender [23], among other attributes. Unfortunately, existing research on the extent of perception of non-racial discrimination in a large population, where data on multiple sources of such discrimination are collected is sparse. As a result, their comparative impacts on health access and outcomes are poorly understood.

Using a representative dataset from one of the most populous and diverse cities in the US, this research investigates whether individuals report experiencing perceived discrimination (henceforth, discrimination) while seeking health care not only due to their race/ethnicity, but also because of their other attributes such as age, gender, type of insurance, and immigration status, the latter group being termed collectively as *non-racial discrimination*. It also empirically examines the association of these two broad categories of discrimination with health access and outcomes.

There are several barriers to producing empirical evidence on different discrimination types. First, self-reported discrimination data have either not been routinely collected or not made readily available. Not many surveys include discrimination questions, and even if they do, such items are restricted to race/ethnicity-related questions, such as the Behavioral Risk Factor Surveillance System (BRFSS) surveys (which has now stopped including the question). Specific discrimination modules in some surveys, such as the ones used in the current study, are not publicly available. Addressing this question is critical, because if an individual does not perceive discrimination for their race or ethnicity, but for their other attributes such as age, insurance status, or income, the impact of the latter on healthcare might still be significantly negative, and a narrower definition of discrimination based solely on race/ethnicity would confound the nature of such relationships.

The findings show that both racial and non-racial discrimination are associated negatively with health access and health outcomes. In particular, in several cases, the magnitude of association for non-racial discrimination is both larger in magnitude and statistically significant. New York has previously been described as an enormous 'city-region' [24], and studying the association of discrimination in health care with health access and outcomes is interesting in its own right. However, these results may be informative for understanding such phenomena in other large, diverse, highly populated metropolitan areas.

## Study data and methods

All data are anonymized. The restricted variable was obtained as part of a Data Use Agreement with the NYC Dept. of Hygiene and Mental Health. Ethical approval was provided by the City University of New York (CUNY) Institutional Review Board (IRB, #2018–0473).

### Data and sample

This study utilized individual-level data from the 2016 New York City Department of Health and Mental Hygiene (DOHMH) Community Health Survey (CHS), which is an annual, stratified random digit-dialed phone survey of approximately 10,000 adult New York City residents accessed on September 21, 2019 [25]. The original survey was based on the nationwide BRFSS survey. The primary areas of interest in the survey are health care discrimination, demographics, health status, health access/insurance, diabetes, cardiovascular health, and mental health. Among these, information on the various types of discrimination in health care conditional on experiencing *any* form of discrimination is not publicly available and has been obtained via a Data Use Agreement approved by the DOHMH and the author's Institutional Review Board. The survey data had very few missing or 'don't know’ responses with the latter coded as missing. For example, for the general health question, only 3 respondents refused, and 86 responded 'Don't know' out of 10,000 respondents. For the discrimination question, 4 respondents refused and 40 answered 'Don't know', less than 0.5%. All data were self-reported.

### Discrimination variables

The question assessing discrimination in the 2016 CHS asked respondents, "Thinking of your experiences trying to get health care treatment in the past 12 months, have you felt you were hassled, made to feel inferior, or discriminated against for any reason?" Available response categories were 'Yes,' 'No,' 'Did not seek health care treatment in the past 12 months,' and 'Don't know.' Additionally, that year, the survey asked a series of follow up questions to respondents who answered 'yes.' They were asked, "What was the reason or reasons you felt discriminated against while trying to get health care treatment in the past 12 months:" and presented with the options Race/ethnicity or skin color, Age, Language, Disability, Bodyweight, insurance status or type, Income level, Religion, Sexual orientation, Gender, Gender identity, Immigration status, and Other reason. Since the primary purpose of this study is to underline the existence and potential role of non-racial discrimination in health care, a categorical variable is created to assess discrimination: no discrimination (base category) vs. racial discrimination vs. non-racial discrimination. Specifically, individuals answering 'No' to the above question is assigned No Discrimination (= 1); individuals citing the reason to be race/ethnicity or skin color is assigned Race-based discrimination (= 2), and individuals citing any other reason listed above (except race) for discrimination is assigned to Non-racial discrimination (= 3); individuals replying ‘Don't Know’ are treated as missing. Therefore, for the respondents in both categories, only the race-based category is considered to make the estimates for non-race based measures more conservative. There are only twenty individuals who responded 'yes' to both race-based and at least one of the non-race based discrimination questions. As a robustness check, I performed the same analysis on the sample that excluded them. The results are very similar, as presented in Table A1 in S1 Appendix and Table A2 in S2 Appendix, respectively.

### Outcome variables

Outcome variables include both health access and health status, including both physical and mental health status, as the discrimination can lead to differentials in access to health care, which may lead to disparities in actual health status [26]. To assess health access, the first question used is the following: "Was there a time in the past 12 months when you needed medical care but did NOT get it? Medical care includes doctor's visits, tests, procedures, prescription medication, and hospitalizations." In this case, coding in the original data is retained: 1 = Yes and 0 = No. In addition, responses to the question "When you are sick or need advice about your health, to which of the following places do you usually go?" The responses are categorized into a binary variable 'Informal Care,' which is coded one for the responses "6 = Alternative health care provider, Family/friend/self/Resources, No usual place and other" and zero for the responses "A private doctor, Non-retail clinic, Urgent Care Center, Hospital ED Retail clinic." To assess health status, individuals were asked: "Would you say in general that your health is: excellent, very good, good, fair or poor?" A binary variable, "Poor General Health," was created by combining the responses fair and poor to one and excellent, very good, good to another. Previous research has shown that discrimination can affect both diabetes and hypertension [9, 14, 27]. These conditions are assessed by the questions: "Have you ever been told by a doctor, nurse or other health professionals that you have hypertension, also called high blood pressure?" and "Have you ever been told by a doctor, nurse or other health professionals that you have diabetes?" respectively, with the answers coded as Yes = 1 and No = 0. Previous research has also shown a significant association between discrimination and mental health outcomes [28]. In the survey, mental health status was assessed by a binary variable that assumed value one if an individual reported being depressed within the past two weeks, based on their responses to the 8-item Patient Health Questionnaire (PHQ8), a validated scale of depression, and zero otherwise [29].

### Control variables

Other variables used in the multivariable analysis include demographic, insurance, education, and income. Five race/ethnicity categories are available in the data—non-Hispanic White, non-Hispanic Black, Hispanic, Asian and Pacific Islanders, and Others. The income-to-poverty ratio in each respondent's household is classified as an income of less than 200% of the federal poverty level and between 200% and 599% of the federal poverty level, and greater than 600% of the federal poverty level. Education is dichotomized as a college graduate and above vs. non-graduate. Likewise, individuals are categorized as having been employed or not.

### Statistical analysis

All statistical analyses use survey weights provided in the NYC CHS data to control for complex survey design. First, the (weighted) prevalence estimates of various categories of self-reported discrimination are calculated to describe the overall prevalence of such a phenomenon. Similarly, weighted averages and prevalence estimates for all the relevant variables and categories are also calculated. Next, to assess the association between discrimination and health access and outcomes, two sets of multivariable logistic regression models are estimated with both racial and non-racial discrimination as predictors of interest (no discrimination being the reference group). In the first model, the primary outcomes of interest correspond to health care access, where the dependent variables are *Denied Care and Informal Care*, respectively. In the second model, those are *Poor Health*, *Depressed*, *High Pressure*, and *Diabetes*, respectively. The individual characteristics in all the regressions included insurance status, immigration status, gender, marital status, employment, race, age group, income-to-poverty ratio, and educational attainment. All regressions have been performed in Stata 15 using survey weights to control for the complex survey design.

## Study results

### Descriptive analysis

Approximately 6% of New York City adults reported experiencing hassles, being made to feel inferior, or being discriminated against for *any* reason while seeking health care treatment during the year prior to the interview (weighted prevalence estimate = 0.57).

**Fig 1:**
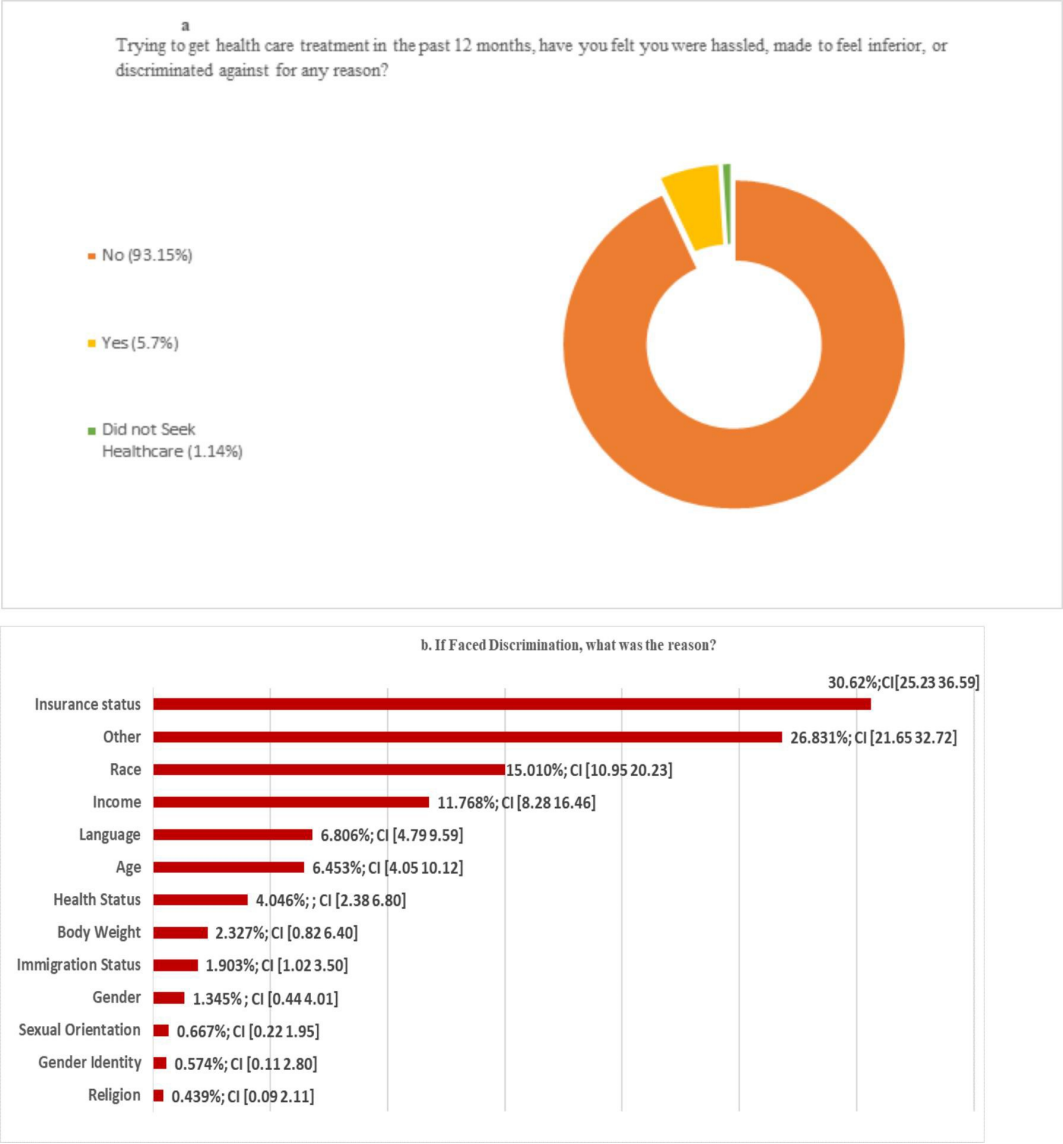
Prevalence and type of discrimination in health care. (A) Trying to get health care treatment in the past 12 months, have you felt you were hassled, made to feel inferior, or discriminated against for any reason? (B) If experienced discrimination, what was the reason?.

Among individuals who experienced discrimination in the past 12 months, 30.62% reported insurance status to be the underlying reason for such discrimination—the highest among all the categories. The next highest category is 'other,' presumably due to the fact that all reasons were not mentioned in the questionnaire (Fig 1). Some other important categories are income, language, age, and immigration status.

Table 1 presents selected prevalence estimates for the sample. The demographic distribution shows the uniqueness of NYC compared to the national population. In the sample, 35% described themselves as non-Hispanic whites (hereafter referred to as white), 22% non-Hispanic black (subsequently referred to as black), 27% Hispanic, 13% Asian or Pacific Islander, and 2% other. Nationally, the composition in 2016 was 61% white, 12% black, 18% Hispanic, 5% Asian, and 1% other groups, including American Indian, Alaska Native, and Hawaii and other Pacific Islanders. The sample is drawn from a population that is otherwise very diverse also. Slightly less than half of the sample (48%) was born in the US, and 35% lived in households where the primary language was not English. In terms of basic demographic profiles, less than half the sample (46%) was male, while less than half (42%) was married. Less than half of the sample (47%) had private insurance, compared to 16% Medicare, 24% Medicaid, 3% other insurance, and 11% uninsured. About a quarter of the households in the sample (26%) reported living below the federal poverty line.

**Table 1:** Summary statistics– 2016 NYC Community Health Survey data.

Variable	Percent
**Race/Ethnicity**	
White Non-Hispanic	35%
Black Non-Hispanic	22
Hispanic	27
Asian/PI Non-Hispanic	13
Other Non-Hispanic	02
**Insurance Status**	
Private	47
Medicare	16
Medicaid	24
Others	03
Uninsured	11
**Age**	
18-24yrs	13
25–44 yrs	40
45–64 yrs	32
65+ yrs	15
**Poverty**	
<100% FPL	26
100 - <200% FPL	22
200 - <400% FPL	17
400 - <600% FPL	16
>600% FPL	19
** = 1 if Male**	46
** = 1 if Married**	42
** = 1 if employed**	60
** = 1 if college graduate**	34
** = 1 if born in US**	52
** = 1 if other language at home**	35

Rows report weighted prevalence rates (expressed in %). FPL: Federal Poverty Level.

SOURCE. Author's analysis of New York City Community Health Survey data for 2016.

### Multivariable analysis

Table 2 reports adjusted odds ratio and confidence intervals from a logistic regression model with two health access variables as outcomes, and race-based discrimination, and non-racial discrimination as two main independent variables. Both models control for insurance status, age, gender, marital status, race/ethnicity, nativity, and poverty. In Table 2, column (1) shows that adults in NYC are almost 7 times more likely not to get needed care when needed when they experience race-based discrimination (AOR = 6.97; 95% CI: [4.15 11.70]) and non-racial discrimination (AOR = 6.96; CI: [5.00 9.70]), respectively.

**Table 2:** Association between health access and types of discrimination: 2016 NYC Community Health Survey.

	(1)	(2)
	1 if Did not get needed care	1 if Medical advice from informal source
Discrimination based on race/ethnicity	6.97***	2.15
	[4.15–11.70]	[0.85–5.43]
Discrimination based on other categories	6.96***	2.24**
	[5.00–9.70]	[1.13–4.48]
**Insurance Ref: Private**		
Medicare	1.66**	0.74
	[1.11–2.47]	[0.38–1.43]
Medicaid	1.85***	1.19
	[1.37–2.51]	[0.67–2.12]
Others	1.77	1.72
	[0.86–3.62]	[0.78–3.80]
Uninsured	2.06***	3.95***
	[1.43–2.96]	[2.29–6.80]
1 if Born in US	0.97	0.68*
	[0.73–1.28]	[0.44–1.04]
1 if Male	1.14	1.75***
	[0.93–1.40]	[1.24–2.48]
1 if Married	0.76**	0.95
	[0.61–0.96]	[0.65–1.38]
1 if college graduate	1.12	1.23
	[0.87–1.43]	[0.80–1.89]
employed	0.96	1.05
	[0.74–1.23]	[0.67–1.65]
1 if Non-English at home	0.76	1.33
	[0.53–1.09]	[0.82–2.18]
**Race Ref: White Non-Hispanic**		
Black Non-Hispanic	0.94	0.87
	[0.69–1.29]	[0.49–1.55]
Hispanic	1.06	0.96
	[0.77–1.45]	[0.58–1.60]
Asian/PI Non-Hispanic	0.84	0.52*
	[0.55–1.27]	[0.24–1.12]
Others	1.32	1.13
	[0.68–2.56]	[0.49–2.64]
**Age Groups Ref: 18-24yrs**		
25–44 yrs	1.44*	0.88
	[0.99–2.08]	[0.51–1.53]
45–64 yrs	1.18	0.49**
	[0.81–1.71]	[0.28–0.87]
65+ yrs	0.71	0.62
	[0.44–1.14]	[0.32–1.20]
**Poverty Groups Ref:<100% FPL**		
100 - <200% FPL	0.99	0.79
	[0.74–1.32]	[0.51–1.23]
200 - <400% FPL	1.14	0.90
	[0.81–1.59]	[0.51–1.56]
400 - <600% FPL	0.93	0.90
	[0.63–1.37]	[0.50–1.60]
>600% FPL	0.61**	0.94
	[0.39–0.97]	[0.46–1.95]
Observations	9,390	9,388

SOURCE. Author's analysis of New York City Community Health Survey data for 2016.

NOTES. Logistic Regression models are estimated using the svy suite of commands in Stata 15, using weights to control for the complex survey design. FPL: Federal Poverty Level. AOR: Adjusted Odds Ratio; 95% Confidence Intervals are in brackets.

*** p<0.01

** p<0.05

* p<0.1

The pattern is similar for seeking informal care, and column (2) reports the point estimates. In this case, individuals experiencing race-based discrimination are 2.15 times more likely to get medical advice from an informal source (AOR = 2.15; CI: [0.85 5.43]), though this particular estimate is not statistically significant. The next row in the same column shows that for individuals experiencing other types of discrimination, the magnitude of the AOR is similar, but the effect is significant at 5% level (AOR = 2.24; CI: [1.13 4.48]. Notably, these results are obtained after controlling for health insurance status, and lack of insurance is independently associated with higher probabilities of either lacking medical treatment or seeking that from informal sources.

The results in Table 3 are from multivariable logistic regressions that estimate the association between three mental and physical health outcomes and two types of discrimination controlling for the same confounding factors as in the previous table. Column (1) shows that independent of the confounding factors, both groups reporting racial and non-racial discrimination were more likely to report poor or worse general health than those not reporting them (AOR = 3.76; CI: [2.15 6.59] and (AOR = 2.49; CI: [1.65 3.75]), respectively. Similarly, column (2) shows that adults who reported these two types of discrimination were more likely to be depressed than their peers (AOR = 6.20; CI: [3.44 11.16] and (AOR = 3.11; CI: [2.02 4.81]), respectively. Results in columns 3 and 4 are both novel and surprising. These two columns report associations of two chronic conditions, hypertension and diabetes, with discrimination. Racial discrimination was associated with an adjusted 144% increase (AOR = 2.44; CI: [1.32 4.51]), and an adjusted 33% increase (AOR = 1.33; CI: [0.59–3.00] in the odds of having been diagnosed with hypertension and diabetes, respectively. The second row of the same columns shows that discrimination was associated with an adjusted 75% increase (AOR = 1.75, CI: [1.21 2.52]) and 84% increase (AOR = 1.84, CI: [1.22 2.77]) in the odds of having been diagnosed with hypertension and diabetes respectively.

**Table 3:** Association between health outcomes and types of discrimination: 2016 NYC Community Health Survey.

	(1)	(2)	(3)	(4)
	1 if General Health Poor	1 if Depressed	1 if has High Pressure	1 if has diabetes
Discrimination based on race/ethnicity	3.76***	6.20***	2.44***	1.33
	[2.15–6.59]	[3.44–11.16]	[1.32–4.51]	[0.59–3.00]
Discrimination based on other categories	2.49***	3.09***	1.75***	1.84***
	[1.65–3.75]	[2.00–4.77]	[1.21–2.52]	[1.22–2.77]
**Insurance Ref: Private**				
Medicare	1.45***	1.47*	1.44***	1.05
	[1.11–1.89]	[0.95–2.28]	[1.13–1.83]	[0.80–1.38]
Medicaid	1.16	1.47**	1.01	0.83
	[0.91–1.48]	[1.03–2.09]	[0.80–1.26]	[0.63–1.09]
Others	1.12	0.88	0.84	0.55**
	[0.68–1.85]	[0.45–1.71]	[0.56–1.27]	[0.31–0.97]
Uninsured	1.10	0.98	0.73*	0.59**
	[0.78–1.55]	[0.60–1.62]	[0.52–1.01]	[0.37–0.94]
1 if Born in US	1.06	1.24	1.22**	0.89
	[0.86–1.32]	[0.92–1.67]	[1.01–1.48]	[0.69–1.14]
1 if Male	0.91	0.91	0.98	1.20*
	[0.77–1.07]	[0.72–1.15]	[0.84–1.14]	[0.99–1.45]
1 if Married	0.94	0.60***	0.87*	1.02
	[0.79–1.13]	[0.47–0.79]	[0.74–1.02]	[0.83–1.25]
1 if college graduate	0.69***	0.49***	0.78***	0.59***
	[0.56–0.85]	[0.37–0.65]	[0.65–0.94]	[0.47–0.74]
employed	0.48***	0.60***	0.68***	0.50***
	[0.40–0.58]	[0.46–0.78]	[0.57–0.81]	[0.40–0.62]
1 if Non-English at home	1.49***	0.85	1.11	0.86
	[1.16–1.91]	[0.59–1.24]	[0.88–1.40]	[0.64–1.15]
**Race Ref: White Non-Hispanic**				
Black Non-Hispanic	0.99	0.54***	2.05***	1.82***
	[0.75–1.29]	[0.37–0.79]	[1.63–2.58]	[1.33–2.48]
Hispanic	1.09	0.83	1.57***	1.99***
	[0.85–1.41]	[0.57–1.20]	[1.23–2.01]	[1.48–2.69]
Asian/PI Non-Hispanic	1.84***	0.50**	0.87	1.45*
	[1.37–2.47]	[0.28–0.86]	[0.65–1.16]	[0.97–2.15]
Others	1.57	0.97	1.23	1.47
	[0.89–2.77]	[0.48–1.97]	[0.71–2.13]	[0.82–2.62]
**Age Groups Ref: 18-24yrs**				
25–44 yrs	2.81***	1.40	2.65***	4.02***
	[1.85–4.29]	[0.91–2.15]	[1.73–4.05]	[1.56–10.34]
45–64 yrs	7.25***	1.91***	11.31***	20.48***
	[4.84–10.86]	[1.26–2.91]	[7.49–17.08]	[8.16–51.43]
65+ yrs	7.45***	1.04	20.06***	30.39***
	[4.88–11.37]	[0.61–1.75]	[13.08–30.76]	[11.88–77.73]
**Poverty Groups Ref:<100% FPL**				
100 - <200% FPL	0.83*	0.64***	0.90	0.81*
	[0.67–1.03]	[0.47–0.88]	[0.71–1.12]	[0.63–1.03]
200 - <400% FPL	0.53***	0.54***	0.91	0.78
	[0.41–0.70]	[0.37–0.78]	[0.71–1.18]	[0.57–1.05]
400 - <600% FPL	0.43***	0.34***	0.74**	0.74*
	[0.31–0.58]	[0.21–0.55]	[0.57–0.97]	[0.53–1.03]
>600% FPL	0.25***	0.25***	0.74*	0.51***
	[0.17–0.37]	[0.15–0.43]	[0.54–1.01]	[0.33–0.79]
Observations	9,358	8,869	9,412	9,416

Logistic Regression models are estimated using the svy suite of commands in Stata 15, using weights to control for the complex survey design. FPL: Federal Poverty Level. AOR: Adjusted Odds Ratio; 95% Confidence Intervals are in brackets.

*** p<0.01

** p<0.05

* p<0.1.

For diabetes, the association with race-based discrimination is not statistically significant, while the one with non-racial discrimination is significant at level 1%. Finally, to check if the above results are sensitive to the choice of models, the models for physical and mental health outcomes were re-estimated by adding three additional control variables—smoking, heavy drinking, and Body Mass Index (BMI). The revised models did not qualitatively alter the findings (for detailed results of the sensitivity analyses, please see Table A3 in S3 Appendix).

### Limitations

Important limitations of these findings include its cross-sectional and self-reported nature. There is a possibility of *ascertainment bias*, which refers to the systematic misrepresentation of the assessment of outcome measures [30]. For example, higher detection of outcome variable like hypertension may be due to higher access to health care, and not necessarily due to higher underlying rates. There is also no information on providers' attitudes or training, though previous research has indicated that many of them might suffer from various forms of bias [17, 31]. The important matter of intersectionality and interaction of various forms of bias was beyond the scope of this study. New York City's unique cultural and demographic factors may have limited generalizability. The methodology also could not necessarily infer a causal link because some unobserved factors such as personal attitudes towards doctors and clinics might influence both discrimination and outcomes like physical and mental health. The perceived discrimination measure suffers from the possibility that people who are unhappy with their experience may sometimes attribute it to discrimination erroneously. However, virtually every study has relied on self-reported data, and given the otherwise absence of evidence, they are useful to policymakers. Additionally, the associations I found of discrimination with health access and outcomes are strong and robust.

## Discussion

In spite of making some progress, the successive goals of eliminating health disparities by the US government in its Healthy People reports have not been met [32]. An extensive literature shows that discrimination in medical settings is a significant determinant of health disparities. However, an overwhelming majority of these studies have focused solely on race/ethnicity as a driver of discrimination, when discrimination can be based on any group membership (gender, immigration, poverty, etc.).

This study empirically investigated the association between self-reported experiences of racial and non-racial discrimination and health access and outcome in one of the most diverse cities in the US. There are two major findings. The first is that in health care settings, the estimated prevalence rate of reporting perceived discrimination due to insurance status is higher than such rates due to race or ethnicity among adults in NYC. Second, though the overall pattern of association between the outcome variables and the two broad categories of discrimination was similar, the magnitudes and significance of estimates were more varied. In some cases, it was the non-racial discrimination that was significantly (and negatively) associated with health outcomes as the corresponding adjusted odds ratios were higher in magnitude and more significant.

There are two possible explanations for these findings. First, when individuals seek health care, they tend to become more vulnerable as patients. If they perceive that they are treated differently from other patients based on some personal attributes such as race, income, insurance, or immigration status, their health status may get affected either directly or indirectly through a lack of future care. Second, while providers may be more sensitive to treating patients of different racial backgrounds, given the prominence of race and ethnicity in discrimination studies and training, they may unconsciously show bias based on other group characteristics.

### Public health policy implications

The results have important implications for addressing the health disparities in New York City and beyond. They underscore why efforts should be made to address *all* forms of discrimination in health care, as those who perceive non-racial discrimination are sometimes at a higher risk for lower health access and outcomes. Public health policymakers should try and collect more comprehensive discrimination data and explore how such information can be combined with interventions like training of health care personnel to reduce adverse impacts on health access and outcomes in the future.

## Supporting information

S1 Appendix Association between health access and types of discrimination: 2016 NYC Community Health Survey–alternative sample (where individuals who perceived both racial and non-racial discrimination in healthcare are excluded). (DOCX)

S2 Appendix Association between health outcomes and types of discrimination: 2016 NYC Community Health Survey–alternative sample (where individuals who perceived both racial and non-racial discrimination in healthcare are excluded). (DOCX)

S3 Appendix Association between health outcomes and types of discrimination: 2016 NYC Community Health Survey–alternative specification (controls for three additional confounding variables). (DOCX)
