# Supplementary material for: Beyond race: Impacts of non-racial perceived discrimination on health access and outcomes in New York City
Source: PLoS One. 2020 Sep 24;15(9):e0239482. doi: 10.1371/journal.pone.0239482 (PMC7514095; doi:10.1371/journal.pone.0239482)
Supplement: S1 Appendix — (DOCX) [file pone.0239482.s001.docx]

**S1: Appendix Table A1:** Association between health access and types of discrimination: 2016 NYC Community Health Survey – Alternative Sample (where individuals who perceived *both* racial and non-racial discrimination in healthcare are excluded.)

|  | (1) | (2) |
| --- | --- | --- |
| VARIABLES | 1 if Did not get needed care | 1 if Medical advice from informal source |
|  |  |  |
| Discrimination based on race/ethnicity only | 7.07*** | 2.12 |
|  | [4.05 - 12.35] | [0.80 - 5.60] |
| Discrimination based on other categories only | 6.97*** | 2.25** |
|  | [5.00 - 9.71] | [1.13 - 4.49] |
| Insurance |  |  |
| Ref: Private |  |  |
| Medicare | 1.62** | 0.73 |
|  | [1.08 - 2.43] | [0.38 - 1.42] |
| Medicaid | 1.84*** | 1.16 |
|  | [1.36 - 2.50] | [0.65 - 2.07] |
| Others | 1.74 | 1.72 |
|  | [0.84 - 3.59] | [0.78 - 3.79] |
| Uninsured | 2.05*** | 3.91*** |
|  | [1.43 - 2.96] | [2.27 - 6.74] |
| 1 if Born in US | 0.97 | 0.67* |
|  | [0.73 - 1.28] | [0.44 - 1.03] |
| 1 if Male | 1.14 | 1.75*** |
|  | [0.93 - 1.40] | [1.24 - 2.49] |
| 1 if Married | 0.76** | 0.94 |
|  | [0.61 - 0.96] | [0.64 - 1.36] |
| 1 if college graduate | 1.11 | 1.21 |
|  | [0.86 - 1.43] | [0.79 - 1.87] |
| employed | 0.96 | 1.03 |
|  | [0.74 - 1.24] | [0.66 - 1.62] |
| 1 if Non-English at home | 0.76 | 1.34 |
|  | [0.53 - 1.09] | [0.82 - 2.19] |
| Race |  |  |
| Ref: White Non-Hispanic |  |  |
| Black Non-Hispanic | 0.94 | 0.87 |
|  | [0.69 - 1.29] | [0.49 - 1.54] |
| Hispanic | 1.06 | 0.96 |
|  | [0.77 - 1.45] | [0.58 - 1.61] |
| Asian/PI Non-Hispanic | 0.84 | 0.52* |
|  | [0.55 - 1.27] | [0.24 - 1.12] |
| Others | 1.29 | 1.02 |
|  | [0.66 - 2.54] | [0.42 - 2.45] |
| Age Groups |  |  |
| Ref: 18-24yrs |  |  |
| 25-44 yrs | 1.42* | 0.87 |
|  | [0.98 - 2.06] | [0.50 - 1.51] |
| 45-64 yrs | 1.16 | 0.49** |
|  | [0.80 - 1.68] | [0.28 - 0.87] |
| 65+ yrs | 0.70 | 0.61 |
|  | [0.43 - 1.14] | [0.32 - 1.19] |
| Poverty Groups | 0.99 | 0.80 |
| Ref:<100% FPL | [0.74 - 1.33] | [0.52 - 1.25] |
| 100 - <200% FPL | 1.14 | 0.91 |
|  | [0.81 - 1.59] | [0.52 - 1.58] |
| 200 - <400% FPL | 0.93 | 0.91 |
|  | [0.63 - 1.37] | [0.51 - 1.62] |
| 400 - <600% FPL | 0.61** | 0.96 |
|  | [0.38 - 0.97] | [0.46 - 1.98] |
| >600% FPL | 0.07*** | 0.03*** |
|  | [0.04 - 0.13] | [0.01 - 0.08] |
|  |  |  |
| Observations | 9,372 | 9,370 |

SOURCE Author’s analysis of New York City Community Health Survey data for 2016

NOTES Logistic Regression models are estimated using the svy suite of commands in Stata 15, using weights to control for the complex survey design. FPL: Federal Poverty Level. AOR: Adjusted Odds Ratio; 95% Confidence Intervals are in brackets. *** p<0.01, ** p<0.05, * p<0.1
