# Supplementary material for: Beyond race: Impacts of non-racial perceived discrimination on health access and outcomes in New York City
Source: PLoS One. 2020 Sep 24;15(9):e0239482. doi: 10.1371/journal.pone.0239482 (PMC7514095; doi:10.1371/journal.pone.0239482)
Supplement: S2 Appendix — (DOCX) [file pone.0239482.s002.docx]

**S2: Appendix Table A2:** Association between health outcomes and types of discrimination: 2016 NYC Community Health Survey – Alternative Sample (where individuals who perceived *both* racial and non-racial discrimination in healthcare are excluded.)

|  | (1) | (2) | (3) | (4) |
| --- | --- | --- | --- | --- |
| VARIABLES | 1 if General Health Poor | 1 if Depressed | 1 if has High Pressure | 1 if has diabetes |
|  |  |  |  |  |
| Discrimination based on race/ethnicity only | 4.10*** | 6.81*** | 2.40*** | 1.36 |
|  | [2.25 - 7.47] | [3.65 - 12.70] | [1.24 - 4.65] | [0.56 - 3.32] |
| Discrimination based on other categories only | 2.49*** | 3.10*** | 1.75*** | 1.84*** |
|  | [1.65 - 3.75] | [2.01 - 4.78] | [1.21 - 2.53] | [1.22 - 2.77] |
| Insurance |  |  |  |  |
| Ref: Private |  |  |  |  |
| Medicare | 1.45*** | 1.48* | 1.44*** | 1.06 |
|  | [1.11 - 1.90] | [0.96 - 2.30] | [1.13 - 1.83] | [0.80 - 1.39] |
| Medicaid | 1.16 | 1.46** | 1.01 | 0.83 |
|  | [0.91 - 1.48] | [1.03 - 2.08] | [0.80 - 1.26] | [0.63 - 1.09] |
| Others | 1.12 | 0.86 | 0.84 | 0.54** |
|  | [0.68 - 1.84] | [0.44 - 1.70] | [0.56 - 1.26] | [0.30 - 0.96] |
| Uninsured | 1.10 | 0.96 | 0.72* | 0.59** |
|  | [0.78 - 1.54] | [0.58 - 1.59] | [0.52 - 1.00] | [0.37 - 0.94] |
| 1 if Born in US | 1.06 | 1.24 | 1.22** | 0.89 |
|  | [0.86 - 1.32] | [0.92 - 1.66] | [1.01 - 1.47] | [0.69 - 1.14] |
| 1 if Male | 0.91 | 0.91 | 0.98 | 1.20* |
|  | [0.77 - 1.07] | [0.72 - 1.14] | [0.84 - 1.14] | [0.99 - 1.45] |
| 1 if Married | 0.94 | 0.60*** | 0.86* | 1.02 |
|  | [0.79 - 1.13] | [0.46 - 0.78] | [0.74 - 1.01] | [0.83 - 1.25] |
| 1 if college graduate | 0.69*** | 0.49*** | 0.78*** | 0.59*** |
|  | [0.56 - 0.86] | [0.37 - 0.65] | [0.65 - 0.94] | [0.47 - 0.74] |
| employed | 0.48*** | 0.60*** | 0.68*** | 0.50*** |
|  | [0.40 - 0.59] | [0.46 - 0.78] | [0.57 - 0.81] | [0.40 - 0.62] |
| 1 if Non-English at home | 1.50*** | 0.86 | 1.11 | 0.86 |
|  | [1.17 - 1.92] | [0.59 - 1.24] | [0.88 - 1.40] | [0.65 - 1.16] |
| Race |  |  |  |  |
| Ref: White Non-Hispanic |  |  |  |  |
| Black Non-Hispanic | 0.99 | 0.54*** | 2.05*** | 1.82*** |
|  | [0.75 - 1.30] | [0.37 - 0.79] | [1.63 - 2.58] | [1.34 - 2.49] |
| Hispanic | 1.10 | 0.83 | 1.57*** | 1.99*** |
|  | [0.85 - 1.41] | [0.57 - 1.20] | [1.23 - 2.01] | [1.47 - 2.68] |
| Asian/PI Non-Hispanic | 1.83*** | 0.50** | 0.87 | 1.44* |
|  | [1.37 - 2.46] | [0.28 - 0.86] | [0.65 - 1.16] | [0.97 - 2.14] |
| Others | 1.59 | 0.98 | 1.20 | 1.47 |
|  | [0.90 - 2.82] | [0.48 - 1.97] | [0.69 - 2.09] | [0.83 - 2.63] |
| Age Groups |  |  |  |  |
| Ref: 18-24yrs |  |  |  |  |
| 25-44 yrs | 2.79*** | 1.41 | 2.68*** | 4.01*** |
|  | [1.83 - 4.26] | [0.92 - 2.18] | [1.74 - 4.12] | [1.56 - 10.32] |
| 45-64 yrs | 7.18*** | 1.93*** | 11.44*** | 20.42*** |
|  | [4.80 - 10.76] | [1.27 - 2.96] | [7.54 - 17.34] | [8.13 - 51.28] |
| 65+ yrs | 7.41*** | 1.04 | 20.32*** | 30.22*** |
|  | [4.86 - 11.31] | [0.61 - 1.77] | [13.20 - 31.27] | [11.81 - 77.32] |
| Poverty Groups |  |  |  |  |
| Ref:<100% FPL |  |  |  |  |
| 100 - <200% FPL | 0.83* | 0.64*** | 0.90 | 0.81* |
|  | [0.67 - 1.03] | [0.47 - 0.88] | [0.72 - 1.13] | [0.63 - 1.03] |
| 200 - <400% FPL | 0.54*** | 0.54*** | 0.92 | 0.78 |
|  | [0.41 - 0.70] | [0.37 - 0.78] | [0.72 - 1.18] | [0.58 - 1.06] |
| 400 - <600% FPL | 0.43*** | 0.34*** | 0.75** | 0.74* |
|  | [0.32 - 0.58] | [0.21 - 0.55] | [0.57 - 0.97] | [0.54 - 1.03] |
| >600% FPL | 0.25*** | 0.25*** | 0.74* | 0.51*** |
|  | [0.17 - 0.37] | [0.14 - 0.43] | [0.54 - 1.01] | [0.33 - 0.79] |
| Constant | 0.10*** | 0.20*** | 0.06*** | 0.01*** |
|  | [0.06 - 0.18] | [0.10 - 0.40] | [0.04 - 0.10] | [0.01 - 0.04] |
|  |  |  |  |  |
| Observations | 9,340 | 8,852 | 9,394 | 9,399 |

SOURCE Author’s analysis of New York City Community Health Survey data for 2016.

NOTES Logistic Regression models are estimated using the svy suite of commands in Stata 15, using weights to control for the complex survey design. FPL: Federal Poverty Level. AOR: Adjusted Odds Ratio; 95% Confidence Intervals are in brackets. *** p<0.01, ** p<0.05, * p<001.
